# An FPGA-Based DDS-Synchronized Quadrature Lock-In Module for Sweep-Field Demodulation in a Single-Beam SERF Magnetometer

**DOI:** 10.3390/s26123850

**Published:** 2026-06-17

**Authors:** Dongjing Zhang, Xiaojian Hao, Rui Jia, Xinying Yu, Yifei Fu, Nengqiang Ma, Zheming Cui

**Affiliations:** State Key Laboratory of Extreme Environment Optoelectronic Dynamic Measurement Technology and Instrument, North University of China, Taiyuan 030051, China; sz202306190@st.nuc.edu.cn (D.Z.); b20230607@st.nuc.edu.cn (X.Y.); sz202306085@st.nuc.edu.cn (Y.F.); sz202406068@st.nuc.edu.cn (N.M.); sz202406082@st.nuc.edu.cn (Z.C.)

**Keywords:** SERF magnetometer, FPGA, digital lock-in amplifier, direct digital synthesizer, quadrature demodulation, sweep-field demodulation, zero-crossing stability

## Abstract

**Highlights:**

**What are the main findings?**

- A shared DDS generates the modulation signal and the I/Q references on the same frequency basis.
- CIC decimation with a reloadable Kaiser-window FIR filter keeps the dispersion zero-crossing stable under different cutoff settings.

**What are the implications of the main findings?**

- The module supports stable working-point determination during single-beam SERF sweep-field operation.
- The FPGA implementation provides online filter adjustment and phase correction without repeatedly changing the DDS phase.

**Abstract:**

Sweep-field operation in a single-beam spin-exchange relaxation-free (SERF) magnetometer requires stable extraction of the dispersion zero-crossing. A frequency mismatch between the modulation signal and the demodulation references, or an unsuitable low-pass filter, can shift this zero-crossing and affect working-point determination. This paper presents a zero-crossing-stability-oriented FPGA quadrature lock-in module for SERF sweep-field demodulation. The module is designed around two requirements of sweep-field operation: maintaining a common frequency basis between the modulation output and the demodulation references, and preserving the dispersion zero-crossing when the low-pass-filter cutoff frequency is adjusted. A shared direct digital synthesizer generates both the sinusoidal modulation output and the I/Q references, keeping the excitation and demodulation signals on the same frequency basis. After quadrature multiplication, CIC decimation and a reloadable Kaiser-window FIR filter are used for low-pass processing. Board-level tests show a 1000.054 Hz spectral peak for a 1000 Hz setting and a loopback amplitude of 0.496 V, close to the ideal 0.500 V baseband amplitude. On the SERF platform, I/Q rotation reduces the quadrature residual ratio from 32.1% to 0.10%. When the FIR cutoff frequency is changed from 3 to 15 Hz, the maximum zero-crossing difference is about 0.58 ms, corresponding to 0.12% of the 2 Hz sweep period. These results show that the module supports stable zero-crossing extraction and working-point determination during sweep-field operation in a single-beam SERF magnetometer.

## 1. Introduction

Ultra-weak magnetic-field measurement is needed in biomagnetic sensing, precision measurement, and quantum sensing. Superconducting quantum interference devices can reach very high sensitivity, but they require cryogenic cooling, which makes the system bulky and difficult to use in compact instruments. Optically pumped magnetometers (OPMs) provide another way to measure weak magnetic fields without cryogenic cooling. In recent years, OPMs and spin-exchange relaxation-free (SERF) magnetometers have been developed for wearable, arrayed, and miniaturized sensing systems [1,2,3,4,5].

The single-beam SERF magnetometer is a simple and practical form of SERF sensor. In this scheme, one laser beam is used for optical pumping and probing at the same time. The transmitted light carries magnetic-field information and is converted into an electrical signal by a photodetector. Because the optical path is simple, the single-beam scheme is easier to combine with compact magnetic shields, field coils, and digital circuits.

Several studies have improved the operation and performance of single-beam SERF magnetometers from different aspects. Liu et al. reported single-beam operation based on transverse magnetic modulation or DC offset, showing that magnetic-field modulation can be used to obtain vector magnetic-field information in a compact configuration [6]. Tang et al. demonstrated high-sensitivity single-beam operation for three-axis magnetic-field measurement, and a later study improved the bandwidth of SERF magnetometers using amplitude-modulated light [7,8]. Other works further extended single-beam SERF operation to triaxial modulation, crosstalk analysis and suppression, and coordinate-system-rotation-based three-axis measurement [9,10,11]. Closed-loop control and response optimization have also been investigated to improve the dynamic response and practical operation of SERF magnetometers [12,13].

These studies show that magnetic-field modulation, synchronous demodulation, and signal-processing parameters are closely related to the final output of single-beam SERF sensors. However, most of these works focus mainly on sensor configuration, sensitivity, bandwidth, crosstalk, or closed-loop response. From the viewpoint of sweep-field demodulation, the lock-in stage itself is usually not treated as the main object of optimization. In particular, the influence of modulation reference frequency consistency and post-demodulation low-pass-filter settings on the dispersion zero-crossing position is rarely evaluated as a dedicated design criterion. This motivates the SERF-oriented digital lock-in module developed in this work, in which reference synchronization, I/Q phase compensation, and low-pass filtering are evaluated directly according to the stability of the sweep-field dispersion zero-crossing.

Before a SERF magnetometer is used for stable measurement, the residual magnetic field around the vapor cell must usually be compensated. A common method is to apply a low-frequency sweep-field and observe the absorption response. When the residual-field changes, the absorption peak shifts within the sweep period. After the operating condition is adjusted, sinusoidal modulation can be applied, and the first-harmonic response can be extracted by synchronous demodulation. The demodulated dispersion curve has a zero-crossing near resonance, which is useful for working-point determination. Therefore, the stability of this zero-crossing is important for sweep-field operation.

In practice, the zero-crossing is affected by both the atomic sensor response and the electronic demodulation chain. Spin polarization, phase delay in the sensor response, optical alignment, and magnetic fields from heating components can all change the measured signal [14,15,16,17]. In the electronic part, the phase relation between the modulation field and the demodulation references is especially important. If the modulation and reference signals are generated from different sources, a small frequency mismatch may accumulate into phase drift during sweep-field operation. In addition, the low-pass filter after quadrature multiplication suppresses high-frequency mixing terms, but it can also change the peak-valley shape and shift the zero-crossing of the dispersion curve.

Digital lock-in amplifiers provide a natural way to extract the first-harmonic response in this situation, and FPGA-based, microcontroller-based, open-source, and software lock-in implementations have been reported for different weak-signal measurement systems [18,19,20,21,22,23,24,25]. However, these implementations are usually evaluated as general-purpose instruments, with emphasis on frequency accuracy, amplitude accuracy, bandwidth, or noise suppression. In single-beam SERF sweep-field demodulation, the quantity used for working-point determination is the zero-crossing of the demodulated dispersion curve. Therefore, the demodulation module should be evaluated not only by conventional lock-in performance, but also by whether it maintains a common frequency basis between modulation and reference signals and preserves the dispersion zero-crossing under different low-pass-filter settings.

In this work, the design is centered on the stable extraction of the dispersion zero-crossing during sweep-field demodulation. A shared DDS generates both the sinusoidal modulation signal and the I/Q references, so that the excitation and demodulation references remain on the same frequency basis. After quadrature multiplication, CIC decimation and a reloadable Kaiser-window FIR filter are used for low-pass processing. The filter and phase compensation settings are evaluated using measured SERF dispersion curves, with zero-crossing displacement used as the key criterion rather than only waveform smoothness or magnitude response.

The contribution of this work is therefore a SERF-oriented integration and experimental evaluation of the demodulation chain for stable working-point determination. The module is tested first by board-level loopback experiments and then on a single-beam SERF magnetometer platform. The results verify three practical effects required in sweep-field operation: same-frequency modulation/reference generation, suppression of residual quadrature leakage after phase compensation, and stable dispersion zero-crossing extraction under different FIR cutoff settings.

## 2. Materials and Methods

### 2.1. Sweep-Field Demodulation in a Single-Beam SERF Magnetometer

**Figure 1:**
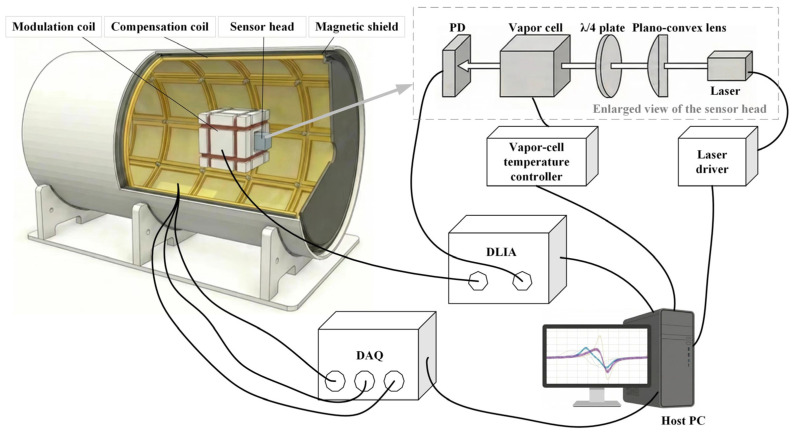
Experimental setup of the single-beam SERF magnetometer. (The colored traces on the host PC monitor schematically represent the results obtained from the same dataset after applying different filtering schemes).

The single-beam SERF magnetometer used in this work is shown in Figure 1. It includes an alkali-vapor cell, a laser, a photodetector, a magnetic shield, compensation coils, and a modulation coil. The laser beam passes through the vapor cell, and the transmitted light is converted into a voltage signal by the photodetector. The compensation coils are used to adjust the magnetic field around the vapor cell. The modulation coil is used to apply the modulation field for lock-in detection. In Figure 1, DLIA denotes the FPGA-based digital lock-in module used in this work.

**Figure 2:**
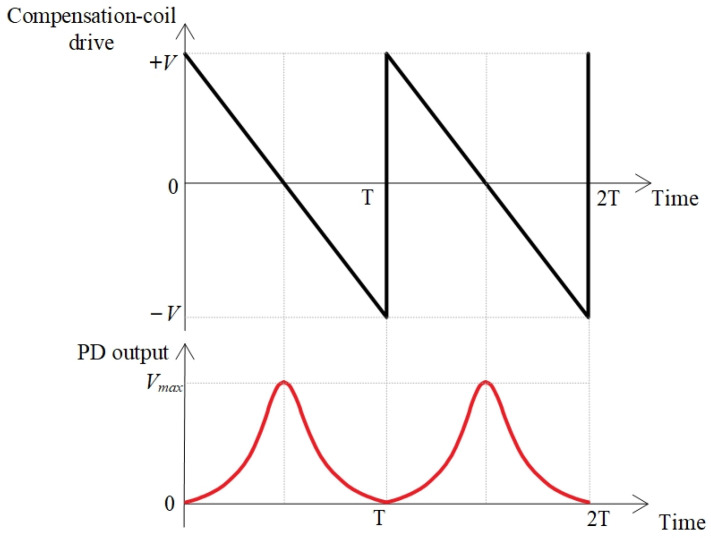
Sawtooth sweep and PD response.

Before lock-in demodulation, a low-frequency sawtooth sweep-field is applied to the compensation coil to scan the resonance region, as shown in Figure 2. Under the near-zero residual-field condition, the absorption peak appears at the expected position in the sweep period. If a residual magnetic field remains, the absorption peak shifts away from this position. Therefore, the absorption peak position can be used to guide residual-field compensation.

**Figure 3:**
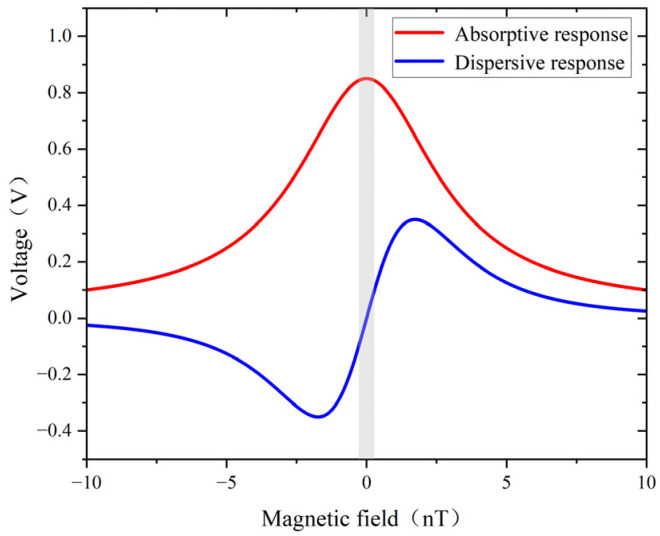
Schematic absorptive and dispersive responses near resonance. (The gray shaded region indicates the near-zero-field linear region of the dispersive response, which is used for zero-crossing-based working-point determination).

After the residual-field is adjusted, a sinusoidal magnetic field is applied through the modulation coil. This modulation produces a first-harmonic component in the photodetector signal. The signal is then demodulated by two orthogonal references and processed by a low-pass filter. The demodulated dispersion curve, shown schematically in Figure 3, has a zero-crossing near resonance. This zero-crossing is used for working-point determination.

### 2.2. FPGA Lock-In Module and DDS-Synchronized Reference Generation

**Figure 4:**
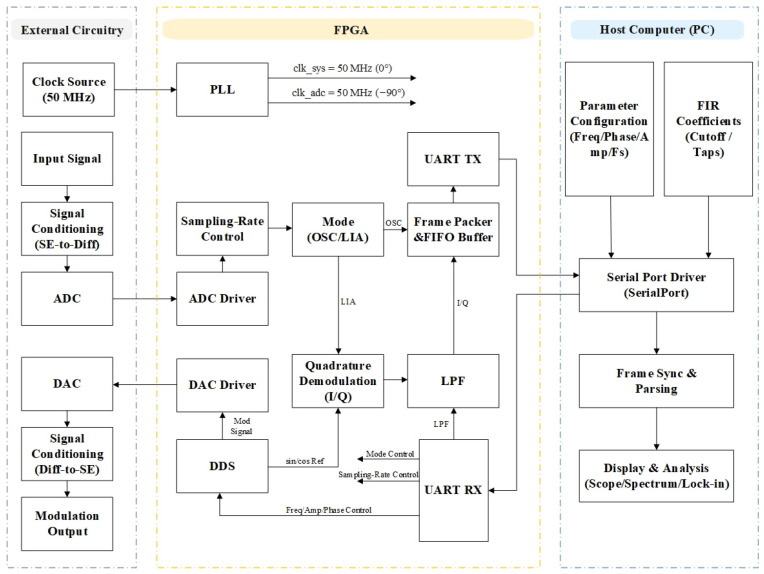
Block diagram of the DDS-synchronized FPGA quadrature lock-in module. The LPF block consists of CIC decimation and a reloadable Kaiser-window FIR filter.

The FPGA lock-in module is shown in Figure 4. It performs signal generation, data acquisition, quadrature demodulation, low-pass processing, and data transmission. The photodetector signal is conditioned, sampled by the ADC, and sent to the FPGA. The FPGA generates the sinusoidal modulation signal through the DDS and DAC path. The DAC output is then conditioned and applied to the modulation coil.

Inside the FPGA, the sampled signal can be sent directly to the host computer for waveform and spectrum observation, or processed by the lock-in path. In the lock-in path, the sampled signal is multiplied by the I/Q references and then filtered by the low-pass stage. The host computer sends modulation, sampling, phase, and FIR-coefficient settings through the serial port. It also receives the output frames and displays the raw waveform, spectrum, I/Q outputs, and lock-in amplitude. In Figure 4, OSC denotes waveform-observation mode, and LIA denotes lock-in mode.

The FPGA platform was used to provide a deterministic timing framework for the complete demodulation chain. In the sweep-field workflow, DDS phase accumulation, DAC waveform output, ADC sampling control, quadrature multiplication, CIC decimation, FIR filtering, and frame transmission are coupled by their timing relationship, which determines the phase consistency between the excitation path and the demodulation references. Implementing these operations in a hardware-parallel FPGA structure keeps the modulation signal and the I/Q references phase coherent during long-term operation and reduces timing uncertainty in the signal path. The same structure also provides a scalable basis for future multi-channel acquisition or closed-loop magnetic-field compensation.

**Figure 5:**
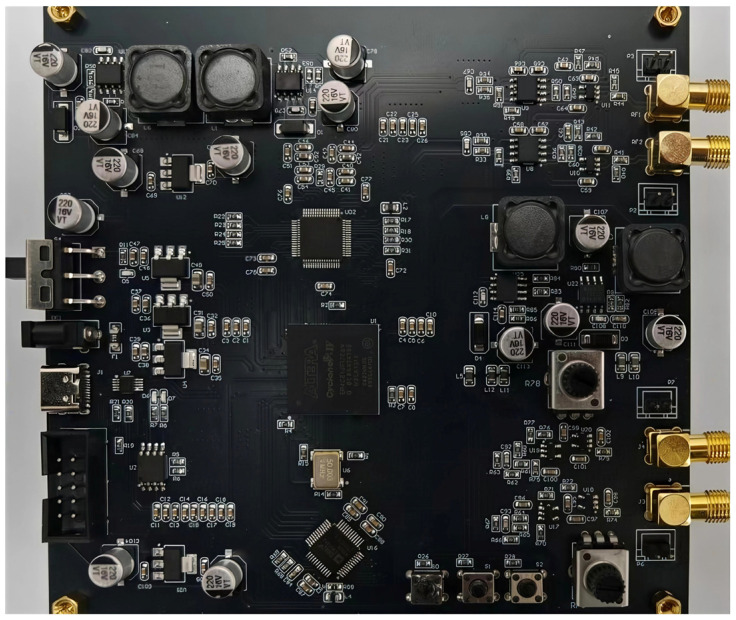
Hardware prototype of the FPGA lock-in module.

The hardware prototype of the FPGA lock-in module is shown in Figure 5. The board integrates the FPGA, ADC input, DAC output, clock circuit, power supplies, signal-conditioning circuits, and communication interface. The conditioned photodetector signal is sampled by the ADC and processed in the FPGA. The DAC output is used to drive the modulation coil after signal conditioning.

The analog input was digitized using an AD9248 dual-channel 14-bit ADC (Analog Devices, Inc., Norwood, MA, USA) with a maximum sampling rate of 65 MSPS. The modulation output was generated by an AD9767 dual-channel 14-bit DAC (Analog Devices, Inc., Norwood, MA, USA) with a maximum update rate of 125 MSPS. In the present experiments, one ADC channel was used for the conditioned photodetector signal and one DAC channel was used for the sinusoidal modulation output; the other channels were reserved for future extension. The 16-bit format listed below refers to the fixed-point FIR coefficients and related digital data formats, not to the ADC or DAC resolution.

A shared direct digital synthesizer (DDS) is used to generate both the sinusoidal modulation signal and the I/Q references. The DDS includes a frequency control word, a phase accumulator, a waveform look-up table, and an amplitude control unit. If the system clock frequency is $f_{\mathrm{clk}}$, the frequency control word is $F_{\mathrm{word}}$, and the phase accumulator width is N, the DDS output frequency is

(1) $$f_{m}=\frac{F_{\mathrm{word}}f_{\mathrm{clk}}}{2^{N}}.$$

The modulation signal and the I reference are generated from the same phase accumulator. The Q reference is obtained by adding a fixed 90° phase offset to the I reference. In this way, the modulation signal and the demodulation references have the same frequency basis, so relative frequency drift between excitation and demodulation is avoided.

The quadrature demodulation outputs are

(2) $$\begin{array}{c} Q=\mathrm{LPF}\left\{s(t)\cos\left(\omega_{m}t+\varphi \right)\right\},\\ I=\mathrm{LPF}\left\{s(t)\sin\left(\omega_{m}t+\varphi \right)\right\}. \end{array}$$

where s(t) is the sampled photodetector signal, $\omega_{m}$ is the modulation angular frequency, φ is the reference phase, and LPF denotes the low-pass stage after quadrature multiplication. The lock-in amplitude is calculated as

(3) $$A=\sqrt{I^{2}+Q^{2}}.$$

### 2.3. Design Requirements and Parameter Selection for SERF Sweep-Field Demodulation

The main parameters were selected according to the sweep-field operation and the digital lock-in signal chain. The modulation frequency was set to 1 kHz to move the response away from low-frequency drift while remaining compatible with the modulation coil, analog front end, and acquisition chain. The default sampling rate was set to 20 kS/s, giving 20 samples per modulation period for the 1 kHz reference. This sampling density is sufficient for quadrature multiplication while keeping the subsequent data rate manageable.

After same-frequency quadrature multiplication, the desired baseband component is accompanied by high-frequency mixing terms around twice the modulation frequency. For the 1 kHz modulation used here, the dominant mixing term appears near 2 kHz. The CIC decimation factor was set to 40, reducing the data rate from 20 kS/s to 500 S/s. The corresponding CIC null spacing is 20 kS/s/40 = 500 Hz; therefore, the 2 kHz mixing term lies near the fourth CIC null before the FIR stage. This relation provides a quantitative reason for the selected decimation factor in addition to data-rate reduction.

For the 2 Hz sweep-field, the 500 S/s FIR input rate corresponds to a 2 ms output interval and about 250 samples per sweep period. This resolution is adequate for displaying the periodic dispersion curve and estimating the zero-crossing position by interpolation near the sign change.

The FIR stage uses a 512-tap Kaiser-window filter with beta = 10 and reloadable coefficients. The default cutoff frequency is 10 Hz, while 3, 5, 10, and 15 Hz settings are compared experimentally to evaluate how low-pass filtering affects the zero-crossing position. Under the 500 S/s FIR input rate and 512-tap configuration, the lowest practical −3 dB cutoff frequency is about 0.9 Hz.

The deterministic delay of the digital low-pass stage was also considered. A 512-tap linear-phase FIR filter at 500 S/s has a group delay of approximately (512 − 1)/(2 × 500) = 0.511 s. Including the CIC decimation delay, the estimated digital low-pass delay is about 0.514 s. In the present sweep-field procedure, the analysis focuses on the steady periodic dispersion waveform and its zero-crossing consistency after filtering, rather than on high-bandwidth feedback latency.

The main hardware and signal-processing parameters are summarized in Table 1.

The FPGA design was compiled using Quartus II 13.0 (Altera Corporation, San Jose, CA, USA). Table 2 gives the resource use reported after compilation. These data are included to show the implementation scale of the module on the selected FPGA device.

### 2.4. Low-Pass Processing and Phase Compensation

After quadrature multiplication, the output contains the desired baseband component and high-frequency mixing terms. A low-pass stage is therefore required before the dispersion curve is used for working-point determination.

In this work, the low-pass stage consists of CIC decimation followed by a Kaiser-window FIR filter. The CIC stage reduces the data rate before FIR filtering. With the default sampling rate of 20 kS/s and a decimation factor of 40, the FIR input rate is reduced to 500 S/s. The FIR filter uses 512 taps and supports coefficient reloading. Therefore, the cutoff frequency can be changed by loading different FIR coefficients, without modifying the FPGA logic.

For sweep-field SERF demodulation, the low-pass filter should not be selected only by waveform smoothness or magnitude response. A lower cutoff frequency can suppress noise, but it may also change the peak-valley shape or shift the zero-crossing of the dispersion curve. In this work, the filter choice is checked using measured SERF dispersion curves, and the comparison is given in the Section 3.

The sensor response, analog front end, and digital processing introduce a phase delay between the modulation field and the demodulated signal. As a result, the useful dispersive component may appear in both I and Q outputs. To reduce this mixing, the filtered I/Q outputs are rotated by an angle θ:

(4) $$\begin{array}{c} I^{\prime }=I\cos\theta +Q\sin\theta ,\\ Q^{\prime }=-I\sin\theta +Q\cos\theta . \end{array}$$

This operation is applied after low-pass filtering. It does not change the DDS output or the preceding quadrature multiplication. Therefore, it can be used for phase adjustment without repeatedly changing the DDS phase.

### 2.5. Experimental Procedure

The module was tested in two steps. First, a board-level loopback test was performed. The DAC output was directly connected to the ADC input. The DDS output was set to a 1000 Hz sinusoidal signal with an amplitude of ±1000 mV. This test was used to check the DDS output frequency, ADC acquisition, quadrature multiplication, and low-pass processing under a controllable electrical condition.

Second, the module was connected to the single-beam SERF magnetometer platform. A 2 Hz sawtooth signal was applied to the compensation coil to scan the resonance region. The absorption peak position was observed and used to adjust the residual magnetic field. After the absorption peak was moved close to the desired position, a 1 kHz sinusoidal modulation field was applied through the modulation coil. The photodetector signal was sampled at 20 kS/s and processed by the FPGA module.

The SERF platform test included three parts. First, the sweep-field absorption response was recorded under different residual-field conditions. Second, the demodulated I/Q outputs were used for phase compensation. Because the sweep-field response is periodic during continuous lock-in operation, one representative complete sweep period before phase compensation and another representative complete sweep period after applying I/Q rotation were selected to compare the relative residual Q component. Third, the FIR cutoff frequency was set to 3, 5, 10, and 15 Hz to compare the dispersion zero-crossing under different low-pass settings. For each cutoff setting, one complete sweep period was selected from the same sweep interval, and the zero-crossing position was obtained by interpolation near the zero-crossing.

## 3. Results

### 3.1. Board-Level DDS Output and Loopback Demodulation

A board-level loopback test was first carried out before the module was connected to the SERF magnetometer. In this test, the DAC output was directly connected to the ADC input. The DDS output was set to a 1000 Hz sinusoidal signal with an amplitude of ±1000 mV. This test was used to check whether the DDS output, ADC sampling, quadrature multiplication, and low-pass processing worked correctly under a controllable electrical condition.

**Figure 6:**
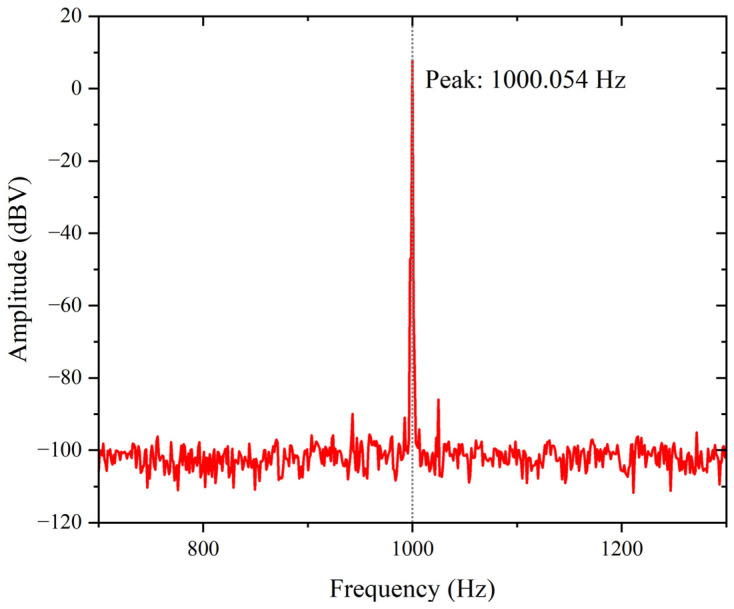
Spectrum of the 1000 Hz sinusoidal output.

As shown in Figure 6, the main spectral peak appears at 1000.054 Hz. The measured frequency is close to the set value of 1000 Hz, and the main component is clearly higher than the spectral background. This result shows that the host-computer parameter setting, DDS frequency control, and DAC output can provide a stable sinusoidal signal for the following demodulation test.

**Figure 7:**
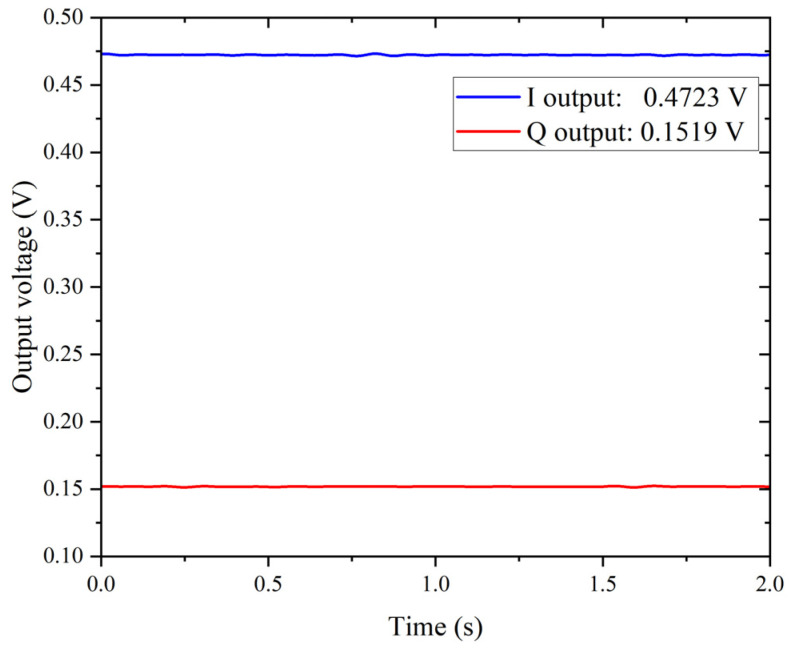
Filtered I/Q outputs under DAC-to-ADC loopback demodulation.

The same loopback signal was then processed by the I/Q demodulation path. The filtered I and Q outputs are shown in Figure 7.

Both I and Q outputs remain at stable DC levels. If there were a relative frequency offset between the input signal and the demodulation references, the low-pass outputs would contain a slowly varying beat component instead of stable DC values. The measured DC values are I = 0.4723 V and Q = 0.1519 V. The resultant amplitude is therefore

(5) $$A=\sqrt{I^{2}+Q^{2}}=0.496\;V.$$

In the loopback test, both the DAC output amplitude and the reference amplitude were set to 1 V. After the multiplication of two same-frequency sinusoidal signals, the baseband term had an ideal amplitude of 0.5 V. The measured amplitude of 0.496 V is close to this value, indicating that the module correctly performs reference generation, quadrature demodulation, and low-pass processing.

### 3.2. Low-Pass Filter Comparison Using Measured SERF Data

The low-pass stage was then examined using measured photodetector data from the SERF platform. The data were obtained under a 2 Hz sweep-field, 1 kHz modulation, and 20 kS/s sampling rate. Two low-pass schemes were compared after CIC decimation: CIC followed by a Butterworth IIR filter, and CIC followed by a Kaiser-window FIR filter.

**Figure 8:**
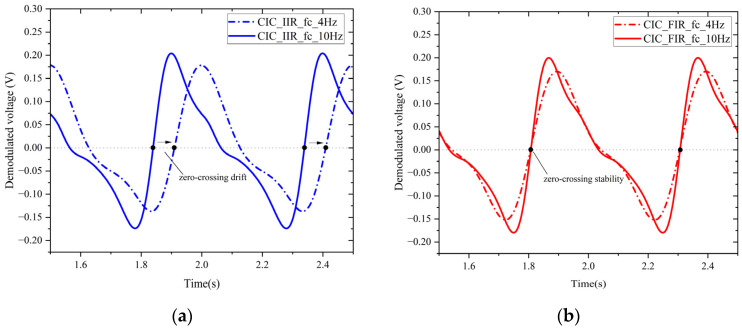
Demodulated dispersion curves with different low-pass schemes: (**a**) CIC + Butterworth IIR; (**b**) CIC + Kaiser-window FIR. (The dotted horizontal lines indicate the zero-voltage baselines used for zero-crossing determination, the black dots mark the zero-crossing points, and the arrows highlight the zero-crossing displacement or stability under different cutoff-frequency settings).

Figure 8a shows that the CIC + Butterworth IIR result changes clearly when the cutoff frequency is reduced from 10 Hz to 4 Hz. The peak and valley positions move, and the zero-crossing also shifts. This means that the filter changes not only the noise level, but also the curve shape used for working-point judgment.

Figure 8b shows the result obtained with the CIC + Kaiser-window FIR scheme. When the cutoff frequency is reduced, the curve becomes smoother and the amplitude changes, but the zero-crossing position remains much more stable. Since the zero-crossing of the dispersion curve is used in sweep-field operation, the CIC + Kaiser-window FIR scheme was used in the following experiments.

### 3.3. Sweep-Field Absorption Response for Residual-Field Compensation

**Figure 9:**
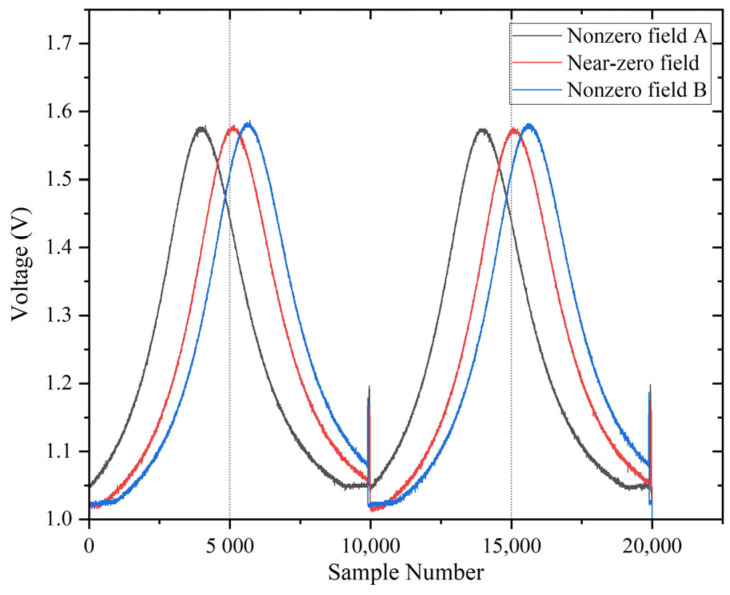
Absorption responses under different residual-field conditions.

After the module was connected to the single-beam SERF magnetometer, a 2 Hz sawtooth voltage was applied to the compensation coil to scan the resonance region. The absorption response was recorded under different residual-field conditions, as shown in Figure 9. The dashed lines mark the midpoint of each sawtooth sweep period.

When a residual magnetic field remains, the absorption peak shifts away from the midpoint of the sweep period. By adding a DC offset to the sawtooth drive, the residual-field can be compensated manually. After compensation, the absorption peak moves close to the midpoint. This result shows that the absorption peak position can be used as a direct reference for residual-field compensation before lock-in demodulation.

### 3.4. Phase-Compensated Dispersion Extraction

**Figure 10:**
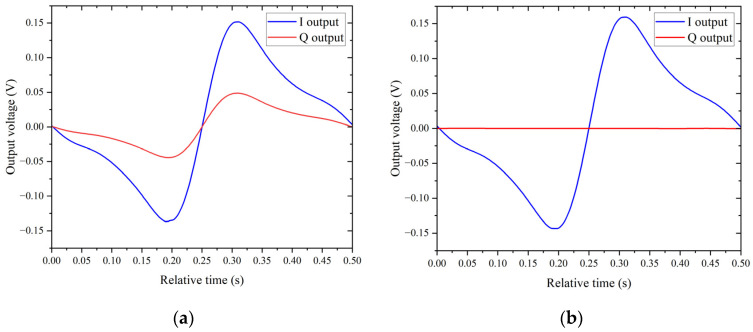
Representative SERF demodulated I/Q outputs: (**a**) before phase compensation; (**b**) after I/Q rotation-based phase compensation.

After the absorption peak was adjusted close to the desired position, a 1 kHz sinusoidal modulation field was applied through the modulation coil, and the photodetector signal was processed by the FPGA lock-in module. The phase compensation effect was evaluated by selecting one representative sweep period before compensation and another representative sweep period after I/Q rotation during continuous operation. Because the sweep-field response is periodic, the time axis in Figure 10 is given as the relative time within each selected sweep period rather than as the absolute acquisition time.

Before phase compensation, the main dispersive response is already observed in the I channel, while the Q channel still contains a visible residual component. After I/Q rotation, the Q component is strongly suppressed and the main response remains in the I channel, indicating that the phase compensation reduces quadrature leakage in the demodulated dispersion output.

In this test, the peak-to-peak value of the selected I-channel dispersion output increases from about 0.2884 V to 0.3026 V. The Q-channel residual decreases from about 0.0925 V to 0.0003 V. The Q-to-I peak-to-peak ratio is reduced from about 32.1% to 0.10%. This result indicates that the rotation-based phase compensation gives a clearer dispersion output for working-point determination.

### 3.5. Cutoff Frequency Adjustment and Zero-Crossing Stability

Based on the phase-compensated SERF data, the cutoff frequency of the CIC + Kaiser-window FIR stage was further adjusted. Four cutoff frequencies were tested: 3, 5, 10, and 15 Hz. For comparison, one complete period of the 2 Hz sweep-field was selected from the same sweep interval for each cutoff setting.

**Figure 11:**
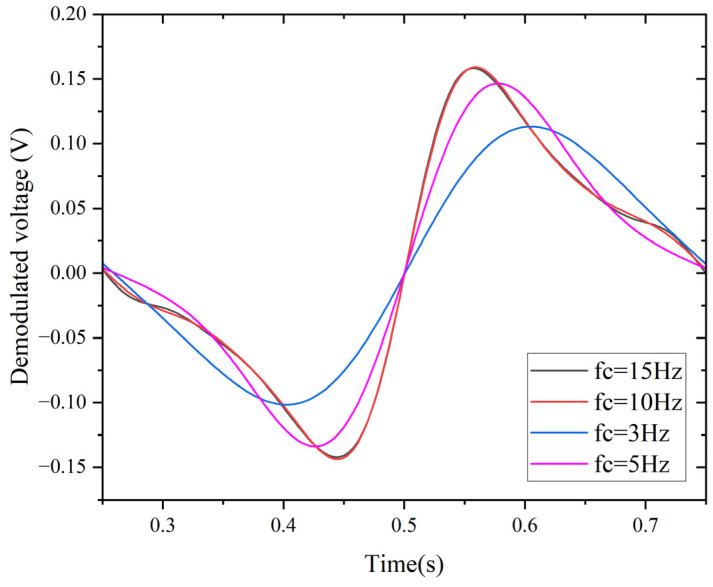
Dispersion curves under different FIR cutoff frequencies.

As shown in Figure 11, changing the cutoff frequency affects the curve amplitude and smoothness. A lower cutoff frequency gives a smoother curve, but also reduces the peak-to-peak amplitude. However, the zero-crossing position remains nearly unchanged. Linear interpolation around the zero-crossing gives a maximum zero-crossing difference of about 0.58 ms among the four cutoff settings. This corresponds to about 0.12% of the 2 Hz sweep period.

These results show that the CIC + Kaiser-window FIR stage can smooth the demodulated signal while keeping the dispersion zero-crossing stable. This is useful for sweep-field SERF operation, where the zero-crossing is used for working-point determination.

The main quantitative results of the board-level and SERF platform tests are summarized in Table 3.

## 4. Discussion

The results demonstrate a SERF-oriented evaluation of the complete demodulation chain for stable sweep-field zero-crossing extraction. In this work, shared DDS generation, CIC decimation, FIR filtering, and I/Q rotation are integrated and evaluated according to the specific requirement of dispersion zero-crossing stability. The board-level loopback test first verifies the basic signal generation and demodulation functions of the module. For a 1000 Hz setting, the measured spectral peak is 1000.054 Hz, corresponding to a frequency error of 0.054 Hz, or 0.0054%. In the loopback demodulation test, the filtered I and Q outputs remain at stable DC levels, indicating that no observable low-frequency beat is introduced between the input signal and the demodulation references. The measured loopback amplitude of 0.496 V is close to the theoretical 0.500 V baseband amplitude.

The low-pass-filter comparison provides a central SERF-specific evaluation in this work. In many digital lock-in systems, the low-pass filter is selected mainly according to noise suppression, bandwidth, or amplitude accuracy [18,19,20,21,22,23,24,25]. For SERF sweep-field demodulation, however, the zero-crossing of the dispersion curve is directly used for working-point determination. The measured SERF data show that the CIC + Kaiser-window FIR scheme keeps the zero-crossing position more stable under different cutoff settings than the CIC + Butterworth IIR scheme, even though the curve amplitude and smoothness change.

The SERF platform results show that the module can be used in the typical adjustment workflow of a single-beam SERF magnetometer. The sweep-field absorption response provides a direct indication of the residual magnetic field around the vapor cell. After the absorption peak is moved close to the midpoint of the sweep period, sinusoidal modulation and quadrature demodulation provide the dispersive response for working-point observation.

The phase compensation result is also useful for practical operation. In the measured data, the main dispersive response appears in the I channel, but a visible residual component remains in the Q channel before compensation. After I/Q rotation, the Q/I peak-to-peak ratio decreases from 32.1% to 0.10%. This result shows that the phase adjustment can concentrate the useful dispersion component into one channel without repeatedly changing the DDS phase.

The present evaluation focuses on module-level quantities that directly determine sweep-field demodulation quality: modulation reference consistency, loopback demodulation accuracy, quadrature residual suppression, and dispersion zero-crossing stability. Magnetometer-level noise and signal-to-noise performance also depend on the vapor cell, optical path, magnetic shielding, coils, and operating point. These system-level quantities can be further evaluated under identical SERF operating conditions when the module is used in closed-loop or biomagnetic measurement configurations.

## 5. Conclusions

This paper presents a SERF-oriented FPGA-based DDS-synchronized quadrature lock-in module for stable sweep-field demodulation in a single-beam SERF magnetometer. The module generates the modulation signal and I/Q references from a shared DDS, performs quadrature multiplication, and applies CIC decimation followed by a reloadable Kaiser-window FIR filter. The design is evaluated with the stability of the sweep-field dispersion zero-crossing as the main criterion, together with board-level synchronization and loopback demodulation tests.

Board-level tests show a 1000.054 Hz spectral peak for a 1000 Hz setting and a loopback demodulated amplitude of 0.496 V, close to the ideal 0.500 V value. On the SERF platform, I/Q rotation reduces the quadrature residual ratio from 32.1% to 0.10%. When the FIR cutoff frequency is changed from 3 to 15 Hz, the maximum zero-crossing difference is about 0.58 ms, corresponding to 0.12% of the 2 Hz sweep period. These results show that the proposed module supports stable sweep-field demodulation and working-point determination.

## Figures and Tables

**Table 1:** Key experimental and implementation parameters.

Parameter	Value or Setting
FPGA device	Cyclone IV E EP4CE10F17C8 (Altera Corporation, San Jose, CA, USA)
System clock	50 MHz
ADC/DAC converters	AD9248 ADC/AD9767 DAC
ADC/DAC resolution	14-bit ADC/14-bit DAC
Sampling rate	20 kS/s by default; configurable from 1 S/s to 60 kS/s
Sweep and modulation frequencies	2 Hz sweep-field; 1 kHz sinusoidal modulation
CIC decimation and FIR rate	Decimation factor 40; FIR input data rate 500 S/s
FIR filter	512-tap Kaiser-window FIR, beta = 10
FIR cutoff settings	10 Hz by default; 3, 5, 10, and 15 Hz tested

**Table 2:** FPGA resource utilization after compilation. (“—” indicates that the corresponding utilization is not applicable or not reported in the compilation report).

Resource	Used/Available	Utilization
Total logic elements	7108/10,320	69%
Total combinational functions	6111/10,320	59%
Dedicated logic registers	3847/10,320	37%
Total registers	3847	—
Total pins	50/180	28%
Total virtual pins	2	—
Total memory bits	348,160/423,936	82%
Embedded multiplier 9-bit elements	26/46	57%
Total PLLs	1/2	50%

**Table 3:** Summary of quantitative results obtained in this work.

Category	Metric	Result
Board-level test	DDS output spectral peak	1000.054 Hz for a 1000 Hz setting
Board-level test	Frequency error	0.054 Hz (0.0054%)
Board-level test	Loopback demodulated amplitude	0.496 V, compared with the theoretical 0.500 V
SERF platform	Q/I peak-to-peak ratio before/after phase compensation	32.1%/0.10%
SERF platform	Maximum zero-crossing difference	0.58 ms
SERF platform	Normalized zero-crossing difference	0.12% of the 2 Hz sweep period
Implementation	FPGA logic element utilization	7108/10,320 (69%)
Implementation	FPGA memory bit utilization	348,160/423,936 (82%)
Implementation	Embedded multiplier utilization	26/46 (57%)
Implementation	Estimated digital low-pass delay	About 0.514 s

## Data Availability

The data presented in this study are available from the corresponding author upon reasonable request.

## Abbreviations

The following abbreviations are used in this manuscript:

ADC	Analog-to-digital converter
CIC	Cascaded integrator-comb
DAC	Digital-to-analog converter
DDS	Direct digital synthesizer
DLIA	Digital lock-in amplifier
FPGA	Field-programmable gate array
FIR	Finite impulse response
IIR	Infinite impulse response
I/Q	In-phase/quadrature
LIA	Lock-in amplification
LPF	Low-pass filter
OPM	Optically pumped magnetometer
OSC	Oscilloscope mode
PC	Personal computer
PD	Photodetector
PLL	Phase-locked loop
SERF	Spin-exchange relaxation-free
UART	Universal asynchronous receiver-transmitter
USB	Universal serial bus
